# Long-term prognostic implications and therapeutic target role of hexokinase II in patients with nasopharyngeal carcinoma

**DOI:** 10.18632/oncotarget.7116

**Published:** 2016-02-01

**Authors:** Meng-Xia Zhang, Yi-Jun Hua, Hai-Yun Wang, Ling Zhou, Hai-Qiang Mai, Xiang Guo, Chong Zhao, Wen-Lin Huang, Ming-Huang Hong, Ming-Yuan Chen

**Affiliations:** ^1^ State Key Laboratory of Oncology in South China, Collaborative Innovation Center for Cancer Medicine, Guangzhou, Guangdong 510060, P. R. China; ^2^ Department of Nasopharyngeal Carcinoma, Sun Yat-Sen University Cancer Center, Guangzhou, Guangdong 510060, P. R. China; ^3^ Department of Pathology, Sun Yat-Sen University Cancer Center, Guangzhou, Guangdong 510060, P. R. China; ^4^ Department of Radiotherapy, Sun Yat-Sen University Cancer Center, Guangzhou, Guangdong 510060, P. R. China

**Keywords:** nasopharyngeal carcinoma (NPC), hexokinase II (HK-II), survival, 3-bromo-2-oxopropionate-1-propyl ester (3-BrOP), therapeutic target

## Abstract

Tumor cells preferentially use anaerobic glycolysis rather than oxidative phosphorylation to generate energy. Hexokinase II (HK-II) is necessary for anaerobic glycolysis and displays aberrant expression in malignant cells. The current study aimed to evaluate the role of HK-II in the survival and biological function of nasopharyngeal carcinoma (NPC). Our study demonstrated that high expression of HK-II was associated with poor survival outcomes in NPC patients. When using 3-BrOP (an HK-II inhibitor) to repress glycolysis, cell proliferation and invasion were attenuated, accompanied by the induction of apoptosis and cell cycle arrest at the G1 stage. Furthermore, 3-BrOP synergized with cisplatin (DDP) to induce NPC cell death. Collectively, we provided that the aberrant expression of HK-II was associated with the malignant phenotype of NPC. A combined treatment modality that targets glycolysis with DDP holds promise for the treatment of NPC patients.

## INTRODUCTION

Nasopharyngeal carcinoma (NPC) is a malignancy that originates from the nasopharyngeal epithelium. In most of the world, the incidence rate is lower than 1 in 100,000. However, it is endemic in southern China, with incidence rates between 15 and 50 in 100,000, and remains one of the most common cancers in China [1–3]. NPC is sensitive to radiotherapy and chemotherapy. However, despite intensive radiotherapy and chemotherapy, 8.4–10.9% of patients still experience tumor recurrence [4, 5], and 15–42% of patients with NPC face distant metastasis after initial treatment [6, 7]. These findings indicate the need for further understanding of the etiology and pathogenesis of this disease.

Enhanced glycolysis is a common characteristic of many malignant tumors [8]. An increasing number of studies have confirmed that glycolysis plays a crucial role in malignant neoplasms [9, 10]. Tumor cells show active glycolysis even under aerobic conditions, which is known as the Warburg effect [11]. Hexokinase is a key glycolytic enzyme that catalyzes the first step in the glycolytic pathway and helps to exhibit the Warburg effect. Hexokinase 2 (HK-II), an isoform of this enzyme, has been found to be overexpressed in various tumors, and the expression levels of HK-II is correlated with poor prognosis [12–14]. Both lonidamine and 3-bromopyruvate (3-BrPA), the HK-II inhibitors used in the early stages of treatment, can effectively inhibit glycolysis but require dosing in the high micromolar range and have limited solubility and biodistribution [15, 16]. As a new HK-II inhibitor, 3-BrOP (3-bromo-2-oxopropionate-1 -propyl ester) has good permeability and stability compared with lonidamine and 3-BrPA, with relatively lower drug concentration required for use [17]. 3-BrOP has shown efficacy against leukemia and lymphoma cell lines [15, 17]. Furthermore, the combination of inhibition of the mTOR pathway with inhibitors of glycolysis has been shown to be synergistically effective against lung cancer and leukemia cells [15, 17, 18]. However, the role of HK-II and its inhibitor in energy metabolism and biological function in NPC is still unclear.

In the current study, we explored the role of HK-II in NPC. We assessed whether HK-II expression was elevated in human NPC tissues and associated with survival outcomes. We investigated the use of 3-BrOP to detect the survival, proliferation and invasion of CNE2 and 5–8F cells under normoxic and hypoxic culture conditions. In addition, we studied the effect of the combination of 3-BrOP and cisplatin in treating NPC.

## RESULTS

### High expression of HK-II is associated with poor prognosis in NPC

**Figure 1:**
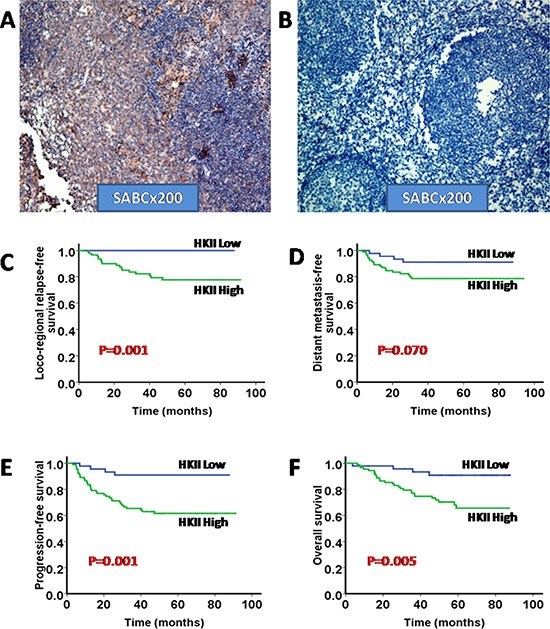
High expression of HKII is associated with poor prognosis in NPC (**A**) Representative micrographs of HKII expression of NPC tissues; (**B**) Representative micrographs of HKII expression of adjacent non-cancerous tissues; Both A and B were shown as labeled (× 200). (**C**–**F**) Kaplan–Meier analysis of the 5-year loco-regional relapse-free survival (LRRFS), distant metastasis-free survival (DMFS), progression-free survival (PFS) and overall survival (OS) regarding the HKII expression levels.

The detailed clinical characteristics of 140 NPC patients are listed in Table 1. Among the 140 patients, 107 (76.43%) were male, and the median age was 48 years (ranged from 19–69 years). The median follow-up time was 52.49 months (ranged from 3.75–93.63 months). The 1-, 3- and 5-year follow-up rates were 99.29%, 93.57% and 84.29%, respectively. The representative pictures for HK-II staining in NPC tissues and adjacent non-cancerous tissues are shown in (Figure 1A–1B). When compared with the HK-II (low expression level) group, the HK-II (high expression level) group experienced significantly shorter LRRFS, PFS and OS (*p* = 0.001, *p* = 0.001 and *p* = 0.005, respectively) and a shorter DMFS with borderline significance (*p* = 0.070) (Figure 1C–1F). A multivariate analysis of the prognostic factors, including age, gender, T stage, N stage, TNM stage, HK-II expression (low/high), was performed. Multivariate analysis indicated that HK-II expression was the independent prognostic factor for PFS and OS (Table 2).

**Table 1:** Correlation of HKII expression with clinical characteristics in 140 patients with NPC

Clinical factor	Cases (*n* = 140)	HK-II expression		*P* Value
		High (*n* = 92)	Low (*n* = 48)	
Sex				0.895
Male	107	70	37	
Female	33	22	11	
Age				0.492
< 50	79	50	29	
≥ 50	61	42	19	
T stage				0.990
T1 + T2	32	21	11	
T3 + T4	108	71	37	
N stage				0.736
N0 + N1	76	49	27	
N2 + N3	64	43	21	
Clinical stage				**0.048**
I + II	12	11	1	
III + IV	128	81	47	
Localregional relapse				**0.001**
Yes	18	18	0	
No	122	74	48	
Distant metastasis				0.062
Yes	23	19	4	
No	117	73	44	
Progression				**< 0.001**
Yes	33	30	3	
No	107	62	45	
Death				**0.003**
Yes	32	28	4	
No	108	64	44	

**Table 2:** Multivariate cox regression analysis

Survival outcome	Variables	Sig	Exp (B)	95% CL for Exp (B)
PFS	Gender	0.645	0.824	0.362–1.874
	Age	0.794	0.907	0.435–1.888
	T stage	0.741	0.879	0.409–1.890
	N stage	0.090	1.618	0.928–2.822
	TNM stage	0.037	3.325	1.072–10.312
	HKII	**0.008**	4.366	1.461–13.049
OS	Gender	0.563	0.797	0.370–1.718
	Age	0.281	0.689	0.349–1.358
	T stage	0.643	0.854	0.437–1.688
	N stage	0.018	1.915	1.116–3.288
	TNM stage	0.258	1.816	0.647–5.100
	HKII	**0.004**	4.845	1.633–14.375

Abbreviations: PFS = progression-free survival; OS = overall survival.

### The best dose point detection of 3-BrOP and CoCl_2_

**Figure 2:**
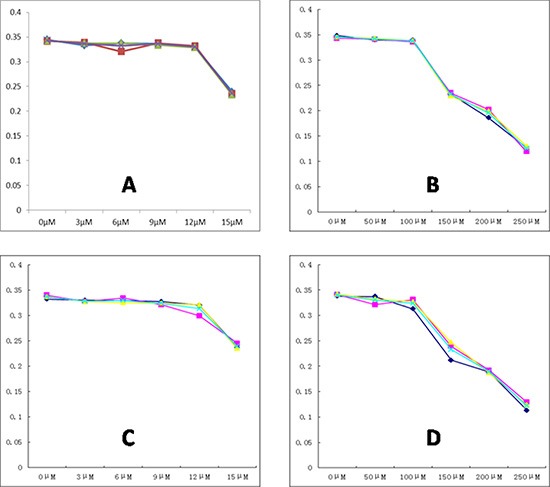
The best dose point detection of 3-BrOP and CoCl2 (**A**) The effect of 3-BrOP treated for 24 h on the growth of CNE2; (**B**) The effect of Cocl2 treated for 24 h on the growth of CNE2; (**C**) The effect of 3-BrOP treated for 24 h on the growth of 5-8F; (**D**) The effect of CoCl2 treated for 24 h on the growth of 5-8F. The best dose point was defined according to the standard curves.

MTT results selected 12 μM as an appropriate working concentration in 5–8F and CNE-2 cells after culturing with 3-BrOP for 24 hours. However, it is preferential to adopt 15 μM in subsequent practice (Figure 2A, 2C). To simulate the hypoxic condition, MTT results recommended incubation with 150 μM CoCl_2_ for 24 hours (Figure 2B, 2D).

### 3-BrOP reduces intracellular ATP concentration of NPC cells

**Figure 3:**
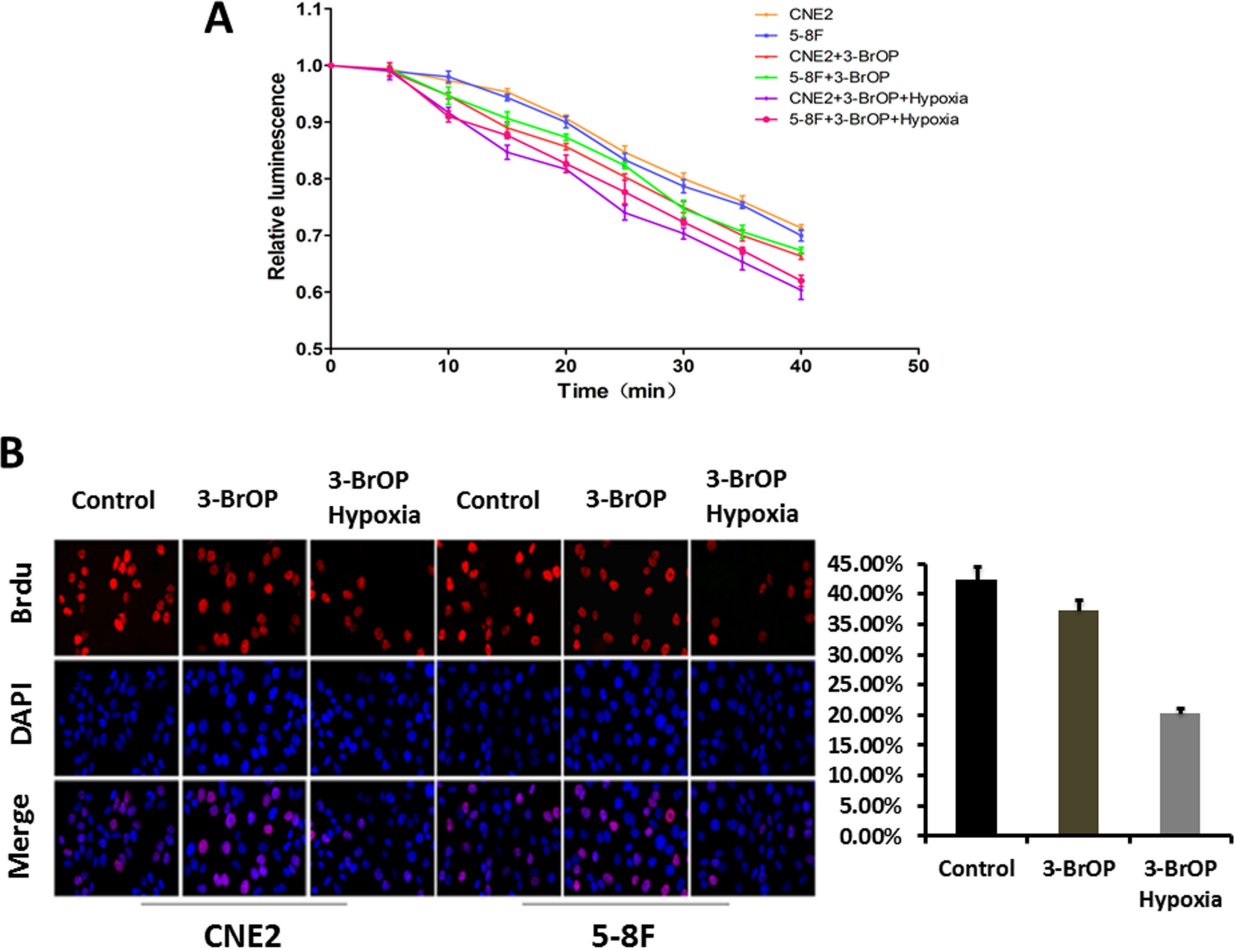
5-8F and CNE-2 cells were cultured with 3-BrOP under hypoxic and normoxic conditions Cells without any treatments were adopted as controls. (**A**) 3-BrOP reduces intracellular ATP concentration of NPC cells. ATP levels were determined using the ATPlite kit according to manufacturer's protocol (PerkinElmer, Boston, MA). (**B**) Growth inhibitory effect of 3-BrOP on NPC cells. The cell proliferation was detected by BrdU in different cell groups.

To determine whether 3-BrOP could induce nonapoptotic necrotic cell death, we detected the intracellular ATP levels of CNE2, CNE2+3-BrOP, 5–8F, 5–8F+3-BrOP under normoxic conditions and CNE2+3-BrOP, 5–8F+3-BrOP under hypoxic conditions, respectively. 3-BrOP was associated with significantly more ATP depletion than control groups in a time-dependent relationship (*p* < 0.05) (Figure 3A). Furthermore, we found a small increase in NPC cell sensitivity to 3-BrOP under hypoxic conditions compared to normoxic conditions.

### Growth inhibitory effect of 3-BrOP on NPC cells

Because the NPC tissues appeared to overexpress HK-II, we next sought to determine whether the inhibition of the factor could inhibit cell growth. A BrdU assay was applied to measure the proliferation ability of NPC cells when treated with 3-BrOP. The results demonstrated that the cell proliferation ability was moderately attenuated in cells treated with 3-BrOP and dramatically attenuated in CNE2+3-BrOP and 5–8F+3-BrOP groups cultured under the hypoxic condition (*p* < 0.05) (Figure 3B).

### 3-BrOP suppresses the invasion ability of NPC cells

**Figure 4:**
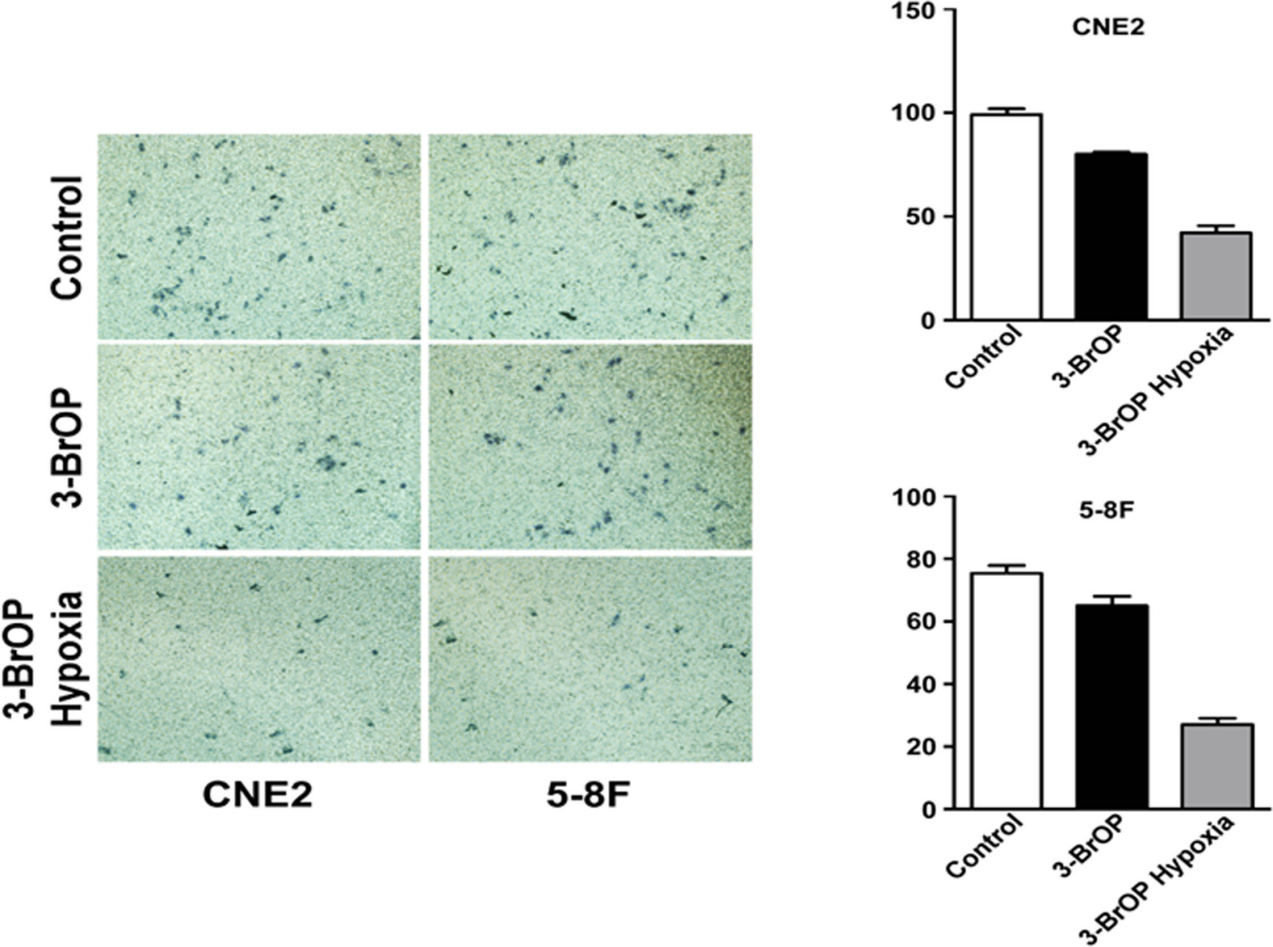
3-BrOP suppresses the invasion ability of NPC cells 5-8F and CNE-2 cells were cultured with 3-BrOP under hypoxic and normoxic conditions. Cells without any treatments were adopted as controls. The cell invasion ability was detected by transwell assay.

A transwell assay was applied to study the invasion ability of NPC cells when treated with 3-BrOP. The invasion ability of CNE-2 and 5–8F were attenuated when cultured with 3-BrOP (*p* < 0.05). The effect was most obvious in the CNE2+3-BrOP and 5–8F+3-BrOP groups cultured under hypoxic conditions. (Figure 4)

### 3-BrOP induces apoptotic cell death and cell cycle arrest at the G1 stage

**Figure 5:**
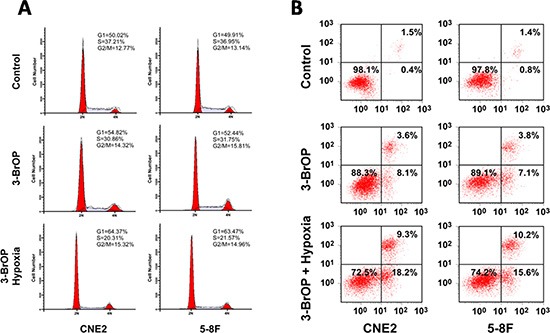
3-BrOP induces apoptotic cell death and cell cycle arrest in G1 stage (**A**) Cell cycles of different groups were detected by flow cytometry; (**B**) Cell apoptosis of different groups were detected by flow cytometry.

We used flow cytometry to detect the cell cycle and apoptosis of NPC cells treated with 3-BrOP. The cell cycle results showed that the cell cycle was arrested at the G1 stage with fewer cells progressing to the S stage. This effect was especially apparent in the CNE2+3-BrOP and 5–8F+3-BrOP groups cultured under hypoxic conditions (Figure 5A). Besides, when cultured with 3-BrOP, the cell apoptosis rate was higher in the treated groups compared with the control group (*p* < 0.05). Similarly, the effect of inducing cell apoptosis was most obvious in the CNE2+3-BrOP and 5–8F+3-BrOP groups cultured under hypoxic conditions (Figure 5B).

### Effect of the combination of 3-BrOP and cisplatin on NPC cells under normoxic and hypoxic conditions

**Figure 6:**
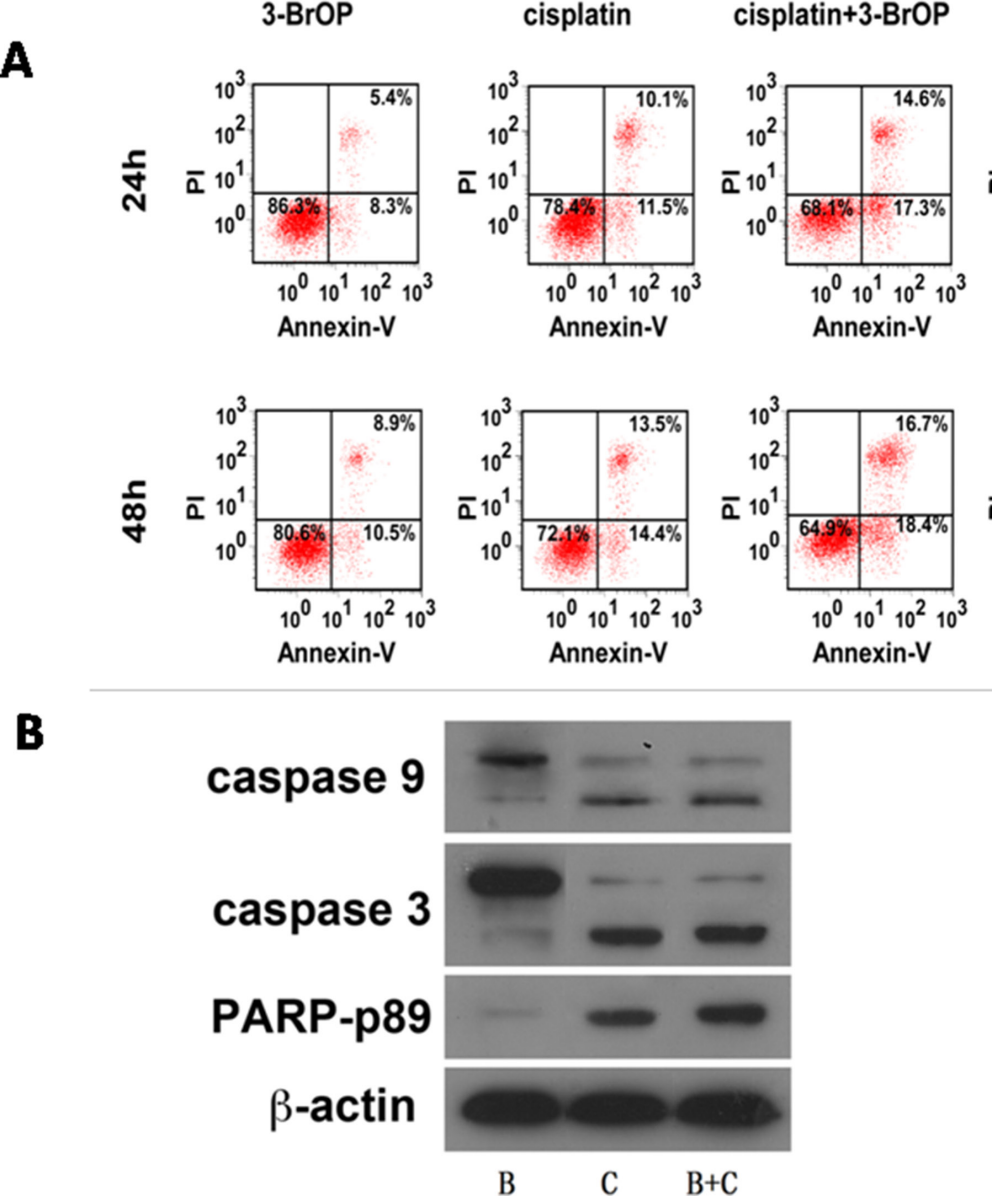
Combination of 3-BrOP and Cisplatin on NPC cells under normoxic and hypoxic conditions (**A**) Apoptosis of CNE2 cells treated with 3-BrOP, cisplatin along and combination; (**B**) Apoptosis-related proteins were detected by western blot.

The development of chemoresistant relapse disease is one of the primary barriers to achieving sustained remission in patients with NPC. Cisplatin remains the standard chemotherapy in NPC. Thus, we further investigated whether 3-BrOP could enhance the cytotoxic effect of cisplatin in NPC cells. CNE-2 and 5–8F were treated with 3-BrOP and DDP alone and in combination. Flow cytometry demonstrated the time-dependent increase of the cell apoptosis proportion in each group. The combination groups demonstrated a higher apoptosis rate than that of the monotreatment groups (Figure 6A). Statistical analyses confirmed that the effect was additive (0.85 < Q < 1.15) [19].

In response to stimuli such as chemotherapy, the intrinsic apoptotic pathway is activated, which results in the activation and cleavage of pro-caspase-9, pro-caspase-3 and pro-PARP [20]. To determine whether the intrinsic apoptotic pathway was activated in response to treatment with cisplatin, HK-II, or combinations thereof, as indicated, an immunoblot analysis of treated cells was performed to determine the activation of caspase-9, caspase-3 and PARP (Figure 6B). As indicated in Figure 6, when combining DDP with 3-BrOP, slight increases in the proteolytic fragments of caspase-9, caspase-3 and PARP were detected.

## DISCUSSION

Increased rates of glycolysis in cancer cells provide an adequate supply of energy and phosphometabolites for biosynthesis, enabling the malignant cells to survive and proliferate even under hypoxic conditions [21, 22]. HKs are key mediators of glycolysis and FDG-uptake in malignant cells. There are four isoenzymes of HK in mammalian tissues designated as type-I, II, III, and IV. Tumors are characterized by the upregulation of HK-I (in brain tumors) and HK-II (in various tumors) [23–24]. Previous studies have confirmed that the expression of HK-II in cancer cells, such as breast, lung, and esophageal cancer cells, is correlated with malignant phenotypes [25, 26]. In this study, we found that high expression levels of HK-II were correlated with poor prognosis, including worse LRRFS, DMFS, PFS and OS compared with low expression levels of HK-II. Multivariate analyses showed that HK-II expression was an independent prognostic factor for PFS and OS. HK-II is often increased in malignant hypoxic cells, thus causing resistance to radiotherapy/chemotherapy and enhanced recurrence and metastasis [27], which may underlie a close relationship between HK-II expression and clinical prognosis.

3-BrOP (3-bromo-2-oxopropionate-1-propyl ester) is a novel inhibitor of glycolysis and has been demonstrated to be efficacious in a variety of preclinical models [15, 17]. Our study represents the first demonstration of the efficacy of hexokinase inhibition in NPC tumor cells. We confirmed the reduction in ATP levels in CNE-2 and 5–8F cells when treated with 3-BrOP. This effect was enhanced when the cells were under hypoxic conditions. Previous studies demonstrated that exposure of neuroblastoma tumor cells to 3-BrOP leads to dramatically decreased ATP concentrations followed by caspase-mediated apoptosis under both normoxic and hypoxic conditions [28]. In addition to the diminished ATP levels, our study confirmed that the apoptosis rate was elevated in cells treated with 3-BrOP and that this effect was partially due to the cell cycle arrest at the G1 stage.

The current study revealed that 3-BrOP can inhibit proliferation and invasion of CNE-2 and 5–8F cells *in vitro*. Previous studies have also confirmed that 3-BrOP-induced depletion of ATP levels was correlated with decreased neuroblastoma cell viability [28]. Detection of cell apoptosis after 3-BrOP exposure suggests that the reduced NPC cell numbers are due at least in part to the induction of apoptosis. Besides, the decreased invasion ability of NPC cells may due part to the suppression of proliferation ability.

The current study showed that combining 3-BrOP with DDP in treating NPC cells can lead to the greatest eradication of tumor cells. This effect was induced by activation of the intrinsic apoptotic pathway. We hypothesized that a significant drop in intracellular ATP may render cells unable to drain out cytotoxic drugs, resulting in increased intracellular concentrations and, ultimately, increased cell death. Based on these promising results, we anticipate that current knowledge and discoveries will be transformed into clinical practice for managing NPC patients in the near future.

### Limitations

First, only two cell lines (CNE-2 and 5–8F) were used to study the cellular function of HK-II. Second, the concentration of 3-BrOP adopted to stimulate NPC cells was unitary. Further study will use concentration gradients of 3-BrOP to observe cell proliferation, invasion and apoptosis. Finally, an *in vivo* experiment was lacking in this study.

## CONCLUSION

The increased dependence of cancer cells on glycolysis offers a rationale for the design of therapeutic strategies to selectively kill cancer cells by inhibition of the glycolytic pathway. HK-II may be a target of therapeutic intervention in cancer cell metabolism. Suppressing the activity of hexokinase may have a beneficial effect on the elimination of NPC cells and may thus improve the treatment outcome.

## MATERIALS AND METHODS

### Tissue specimens and clinicopathological data

The study material consisted of 140 specimens of diagnosed and non-disseminated NPC treated in Sun-Yat Sen University Cancer Center between 2004 and 2008. All specimens were retrieved from the archives of the Tissue Bank. Clinicopathological data including age, gender, T stage, N stage, TNM stage and survival information were gathered. Written informed consent was given to the sample donors, and approval was granted by the Institute Research Medical Ethics Committee of Sun Yat-Sen University.

### Immunohistochemistry

Immunohistochemistry (IHC) was performed to examine HK-II expression in NPC tissues. Formalin-fixed paraffin-embedded (FFPE) tissue blocks were cut into 4-μm sections for IHC, as well as hematoxylin and eosin staining. Polyclonal rabbit anti-HK-II antibody (Thermo Scientific, Waltham, MA, USA) diluted to 1:150 was used in this study. Briefly, tissue sections were de-waxed in xylene and rehydrated through a graded ethanol series, incubated in retrieval buffer solution for antigen recovery, incubated with hydrogen peroxide for 10 minutes to block intrinsic peroxidase and then washed in PBS, blocked with normal serum for 10 minutes, and incubated with a primary antibody for 40 min at 37°C and EnVision^™^ Detection systems (DAKO, Denmark) for 30 min at 37°C. Visualization was developed with diaminobenzidine (DAB). Negative controls were carried out by substituting non-immune rabbit serum for the primary antibodies.

The IHC results were evaluated and independently scored by a pathologist without knowledge of the patient's clinicopathological outcomes. For staining intensity, the score was classified as 0–3 (no staining, weak staining, moderate staining and high staining, respectively). For positive rate, the score was classified as 0–4 (no staining, < 10%, 10%–50%, 50%–80% and > 80%, respectively). The total score was calculated by multiplying these two scores together. Then, the total scores were categorized as no/low expression (≤ 3) and high expression (> 3) [29].

### Cell culture

Human NPC cell lines (5–8F and CNE-2) were cultured in DMEM (Invitrogen) with 10% FBS (HyClone). These cell lines were incubated in a humidified chamber with 5% CO_2_ at 37°C. CoCl_2_ was added to NPC cells for 24 hours to create a hypoxic culture condition and hypoxia-related protein HIF-1αwas detected to see whether hypoxia was induced by CoCl_2_.

### Therapeutic agents

3-bromo-2-oxopropionate-1-propyl ester (3-BrOP) was generously provided by Peng Huang (M.D. Anderson Cancer Center, Houston, TX). A stock solution of 1 M was generated in 1-propanol, stored at 4°C, and diluted in fresh media immediately before use. DDP was dissolved in DMSO to create a 10 mM stock solution stored at −20°C.

### Western blotting

Briefly, cells were ruptured by RIPA buffer (150 mM NaCl, 0.5% EDTA, 50 mM Tris, 0.5% NP40) and kept on ice for 30 minutes, then collected and centrifuged for 25 min at 12000 rpm (4°C). Fifty micrograms of harvested total protein was loaded, separated in 10% sodium dodecyl sulfate-poly-acrylamide gradient gels and transferred onto PVDF membranes followed by blocking with 5% non-fat milk for 2 hours at room temperature. Membranes were incubated with primary antibody and horseradish peroxidase-conjugated secondary antibody and then detected using the ECL chemiluminescence system (Pierce, Rockford, USA).

### MTT

The most effective dose points of 3-BrOP and CoCl_2_ were calculated by the 3-(4, 5-dimethylthiazol-2-yl)-2, 5-diphenyltetrazolium bromide (MTT) assay. Cells were plated in 96-well microplates (6 wells per group) at 3 × 10^3^ cells per well in 200 μl of DMEM complete medium. After stimulating CNE-2 and 5–8F cells using 3-BrOP (0, 3, 6, 9, 12, 15 μM) and CoCl_2_ (0, 50, 100, 150, 200, 250 μM) for 24 hours, 20 μl of MTT reagent (5 mg/ml in PBS) was added to each well and incubated for 4 h. Subsequently, the MTT solution was removed, 150 μl of dimethyl sulfoxide was added to each well and the plate was agitated for 10 min to dissolve the formazan crystals. Absorbance was recorded on a microplate reader at a wavelength of 490 nm. The best dose point was defined according to the standard curves.

### ATP assay

5–8F and CNE-2 cells were plated and treated with the indicated agents. ATP levels were assessed using the ATPlite kit according to the manufacturer's protocol (PerkinElmer, Boston, MA).

### BrdU assay

BrdU assay was performed using the BrdU Cell Proliferation assay kit (Merck, Marmstadt, Germany) following the manufacturer's protocol. Briefly, cells in a 96-well plate were cultured in a medium with BrdU for 12 h. BrdU incorporation was detected with anti-BrdU. Signals were measured by spectrophotometer analysis at 450/540 nm.

### Cell cycle analysis

CNE-2 and 5–8F cells were both classified into 3 groups, CNE2, CNE2+3-BrOP, CNE2+3-BrOP under hypoxic conditions and 5–8F, 5–8F+3-BrOP, 5–8F+3-BrOP under hypoxic conditions, and cultured in 6-well plates until 70%–80% confluent. The used concentrations of 3-BrOP and CoCl_2_ were 12 μM and 150 μM. Cells were then washed with PBS, fixed in ice-cold 70% ethanol and stained with PI buffer (0.1% Triton X-100, 0.2 mg/ml RNaseA, and 0.05 mg/ml PI) for 30 min. The samples were subjected to flow cytometry (BD Biosciences) for cell cycle analysis. The ModFit LT V4.0 software package (Verity Software, Topsham, ME) was used to analyze the data.

### Apoptosis assay

Annexin V-staining was performed using Annexin V-FITC apoptosis detection kit (BD Biosciences, CA, USA) following the instructions of the manufacturer. Briefly, after incubation, cells were harvested, washed with PBS, centrifuged, and stained with Annexin V-FITC and 5 μg/ml propidium iodide in binding buffer (10 mM Hepes, pH 7.4, 140 mM NaCl, 2.5 mM CaCl_2_) for 15 min at 37°C in the dark. The samples were analyzed by flow cytometry using a FACS can flow cytometer. CellQuest software was used to analyze the data (Becton-Dickinson).

### Transwell invasion assay

For the transwell invasion assay, 1.0 × 10^4^ cells in 400 μl of serum-free DMEM were added to the cell culture inserts with an 8-μm microporous filter with extracellular matrix coating (Becton Dickinson Labware, Bedford, MA). The DMEM medium containing 10% FBS was added to the bottom chamber. After 24 hours of incubation, the cells in the lower surface of the filter were fixed and stained followed by microscopic examination. The number of invading cells in three random optical fields (× 100 magnification) for each filter from triplicate inserts was averaged.

### Statistics

The chi-square test was used to evaluate the relationship between HK-II expression and clinical features. The loco-regional relapse-free survival (LRRFS), distant metastasis-free survival (DMFS), progression-free survival (PFS) and overall survival (OS) were calculated using the Kaplan-Meier method and compared using the log-rank test. Durations were calculated from the date of diagnosis to the date of event occurrence or date of last follow-up. Student's *t*-test or Mann-Whitney *U* test was employed to compare the values between subgroups. *P*-values less than 0.05 were considered significant in this study. Analyses were performed using SPSS 18.0 (Chicago, IL, USA).

## SUPPLEMENTARY MATERIALS FIGURES


