# Supplementary material for: Long-term prognostic implications and therapeutic target role of hexokinase II in patients with nasopharyngeal carcinoma
Source: Oncotarget. 2016 Feb 1;7(16):21287–97. doi: 10.18632/oncotarget.7116 (PMC5008285; doi:10.18632/oncotarget.7116)
Supplement: Supplementary file 1 [file oncotarget-07-21287-s001.pdf]

Long-term prognostic implications and therapeutic target role of hexokinase II in patients with nasopharyngeal carcinoma

Supplementary Materials

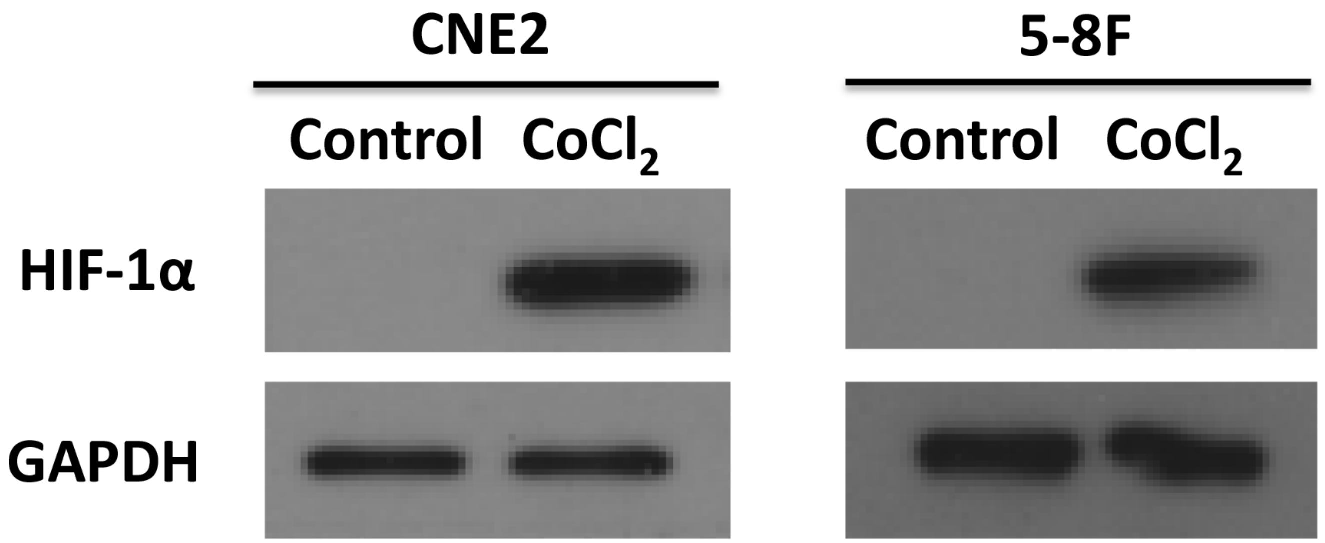

Supplementary Figure S1: The expression of HIF-1a in cells treated with the best dose point concentration of $CoCl_2$ and control groups (same volume of PBS was added). Wb showed that the best dose point concentration of $CoCl_2$ can successfully induce hypoxia by detecting HIF-1α expression.

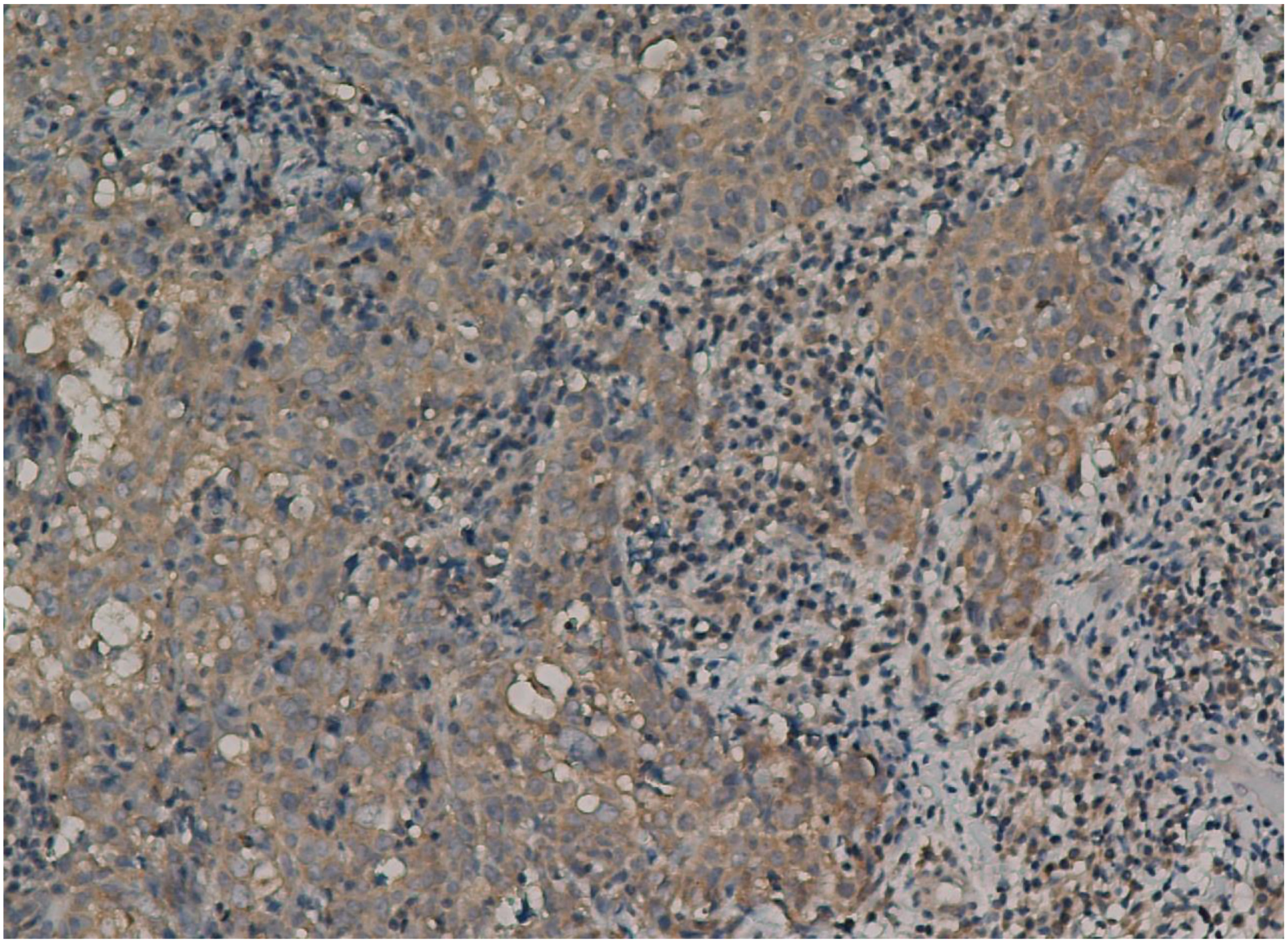

Supplementary Figure S2: The expression of HK-II in NPC tissues (200X). The IHC result demonstrated the location of HK-II in cytoplasmic of NPC tissues.
